# Effects of ultrasound-guided regional anesthesia in cardiac surgery: a systematic review and network meta-analysis

**DOI:** 10.1186/s12871-022-01952-7

**Published:** 2022-12-29

**Authors:** Burhan Dost, Alessandro De Cassai, Eleonora Balzani, Serkan Tulgar, Ali Ahiskalioglu

**Affiliations:** 1 Department of Anesthesiology and Reanimation, Faculty of Medicine, Ondokuz Mayis University, Kurupelit, Samsun, TR55139 Turkey; 2 UOC Anesthesia and Intensive Care Unit, University Hospital of Padua, Padua, Italy; 3 Department of Surgical Science, University of Turin, Turin, Italy; 4 Department of Anesthesiology and Reanimation, Samsun Training and Research Hospital, Samsun University Faculty of Medicine, Samsun, Turkey; 5 Department of Anesthesiology and Reanimation, Ataturk University School of Medicine, Erzurum, Turkey; 6 Clinical Research, Development and Design Application and Research Center, Ataturk University School of Medicine, Erzurum, Turkey

**Keywords:** Cardiac surgery, Meta-analysis, Regional Anesthesia, Ultrasound

## Abstract

**Background:**

The objective of this systematic review and network meta-analysis was to compare the effects of single-shot ultrasound-guided regional anesthesia techniques on postoperative opioid consumption in patients undergoing open cardiac surgery.

**Methods:**

This systematic review and network meta-analysis involved cardiac surgical patients (age > 18 y) requiring median sternotomy. We searched PubMed, EMBASE, The Cochrane Central Register of Controlled Trials (CENTRAL), Scopus, and Web of Science. The effects of the single-shot ultrasound-guided regional anesthesia technique were compared with those of placebo and no intervention. We conducted a risk assessment of bias for eligible studies and assessed the overall quality of evidence for each outcome.

**Results:**

The primary outcome was opioid consumption during the first 24 h after surgery. The secondary outcomes were pain after extubation at 12 and 24 h, postoperative nausea and vomiting, extubation time, intensive care unit discharge time, and length of hospital stay. Fifteen studies with 849 patients were included. The regional anesthesia techniques included pecto-intercostal fascial block, transversus thoracis muscle plane block, erector spinae plane (ESP) block, and pectoralis nerve block I. All the regional anesthesia techniques included significantly reduced postoperative opioid consumption at 24 h, expressed as morphine milligram equivalents (MME). The ESP block was the most effective treatment (-22.93 MME [-34.29;-11.56]).

**Conclusions:**

In this meta-analysis, we concluded that fascial plane blocks were better than placebo when evaluating 24 h MMEs. However, it is still challenging to determine which is better, given the paucity of studies available in the literature. More randomized controlled trials are required to determine which regional anesthesia technique is better.

**Trial registration:**

PROSPERO; CRD42022315497.

**Supplementary Information:**

The online version contains supplementary material available at 10.1186/s12871-022-01952-7.

## Introductıon

Cardiovascular diseases are prevalent in the general population globally and affect most of the older adult population. With the increase in longevity in recent years, there has been a considerable increase in surgical procedures related to cardiovascular diseases [1]. The Society for Enhanced Recovery after Cardiac Surgery (ERAS® Cardiac) recommends effective perioperative pain control to improve patient outcomes. The goals of pain management are to alleviate suffering, gain early mobilization after surgery, reduce hospital stay, and improve patient satisfaction and functional recovery [2].

The pain is most intense during the first two days after cardiac surgery and subsequently decrease [3]. Inadequate acute postoperative pain control after cardiac surgery may result in chronic pain, which affects the quality of life [4]. Seventeen percent of patients report chronic pain one year after cardiac surgery [5]. Inadequate acute postoperative pain control can also increase pulmonary complications due to the inability to breathe, cough, and clear secretions [3]. Response to pain and stress caused by cardiac surgery increases endogenous catecholamines. In addition, tachycardia and hypertension that may occur due to pain during surgery also increase the oxygen requirement of the myocardium. While this condition can be tolerated in normal individuals, it causes ischemia in patients with coronary artery disease, failure in patients with left ventricular dysfunction, or arrhythmias in an electrically unstable myocardium [6]. The leading causes of pain in cardiac surgery procedures are sternotomy/thoracotomy incisions, chest retraction, internal mammary artery harvesting, chest tubes, sternal wires, and visceral pain [7]. Sternal pain is transmitted by the intercostal nerves raised from the T2-T6 spinal nerve roots [8]. The mechanism of cardiovascular surgical pain can be represented as neuropathic and somatic pain, as it is commonly identical to postoperative pain.

Opioids are referred to as anti-ischemic or preconditioning agents with their cardioprotective responses [9]. However, using multimodal opioid-sparing perioperative pain management strategies in current anesthesia practices is recommended instead of systemic analgesics, opioids, or locoregional techniques alone. In addition to pharmacological therapies, regional anesthesia (RA) interventions should be considered for every patient. The limited use of neuraxial procedures or paravertebral block in cardiac surgery with potential hemodynamic instability, full heparinization, and hemodilution is challenging for anesthesiologists [10] Chest wall fascial plane blocks are increasingly used to provide postoperative pain relief and decrease opioid consumption in patients undergoing cardiac surgery and show good results with fewer side effects when compared to central blocks, such as thoracic epidural analgesia or systemic analgesia, considering patients at high cardiovascular risk. In recent years, the development of new RA techniques, due to the role of ultrasonography, has enabled several new fascial plane blocks [11]. Fascial plane blocks are often technically more accessible and less invasive than neuraxial analgesia for cardiac surgery. Several randomized controlled trials have compared the associations between regional anesthesia techniques and postoperative opioid consumption, pain scores, and complications, but the results are inconsistent for cardiac surgery [12]. In addition, there are not enough studies comparing the effects of different fascial plane blocks in this subset of patients; hence, it would be of relevance to examine this aspect.

We hypothesized that the use of single-shot RA techniques would be associated with superior pain control and reductions in 24-h postoperative opioid consumption compared with placebo or systemic analgesics alone. This systematic review and network meta-analysis (NMA) aimed to compare the effects of single-shot ultrasound-guided RA techniques on open cardiac surgery.

## Methods

We pre-registered the protocol on a register (PROSPERO; CRD42022315497), and the manuscript was prepared according to the Preferred Reporting Items for Systematic Reviews and Meta-Analysis (PRISMA) Statement Guidelines [13].

### Eligibility criteria

To determine study eligibility, we used the following PICOS criteria: cardiac surgical patients (age > 18 y) requiring median sternotomy (P), single-shot ultrasound-guided RA technique (I), placebo or no intervention (C), 24-h postoperative opioid consumption, pain after extubation 12 h, pain after extubation 24 h, postoperative nausea and vomiting (PONV), extubation time, intensive care unit discharge time, hospital length of stay (LOS) (O), and randomized controlled trials (RCTs) (S).

Studies were excluded if they met the following additional criteria: 1) the use of RA techniques in combination, 2) the use of continuous RA techniques, 3) minimally invasive cardiac surgery, and 4) off-pump cardiac surgery.

### Search strategy

We performed a systematic search of the medical literature for the identification, screening, and inclusion of articles in the following databases: PubMed, EMBASE, The Cochrane Central Register of Controlled Trials (CENTRAL), Scopus, and Web of Science (last search update April 15, 2022) by two authors (BD and EB) without any restrictions on the language, status, and year of publication. The snowball method was used to review the references included in other studies. Specific information regarding our search strategy is provided in the Supplementary Material (Supplementary material 1).

### Study selection

Initial screening of titles and abstracts was independently performed by two authors (AA and ST) to select relevant and irrelevant manuscripts, and in case of disagreement, a third author (BD) mediated the discussion.

### Data extraction and data retrieval

After identifying the studies that met the inclusion criteria, all the authors manually reviewed and assessed each of the included studies; however, all the extracted data were manually checked by at least two authors.

All opioids were converted to intravenous morphing using the GlobalRPh morphine equivalent calculator, considering a 25% cross-tolerance modifier (http://www.globalrph.com/narcotic).

### Quality assessment and certainty of evidence assessment

Two researchers independently evaluated the quality of included RCTs by using the Risk of Bias (RoB) 2 Tool [14]. The RoB2 tool uses a three-point scale, including “low risk of bias,” “some concerns,” and “high risk of bias,” by investigating five domains at risk of bias. Disagreements were resolved by discussion; if no agreement was reached after discussion, a third researcher (BD) was involved to correctly assign the risk of bias.

The grading of recommendations assessment, development, and evaluation (GRADE) system was used to rate the certainty and, therefore, the quality of evidence for each outcome [15].

### Statistical methods

We used R version 4.1 (R Foundation for Statistical Computing, Vienna, Austria) and the Facenetmeta package to perform the data meta-analysis.

The treatment effect on continuous outcomes was expressed as the mean difference (MD) with 95% confidence interval (CI). The treatment effect for dichotomous outcomes was expressed as an odds ratio (OR) with a 95% CI. The methods were ranked based on the frequentist analog of the surface under the cumulative ranking curve (SUCRA) [16]. The SUCRA assigns numbers ranging from 0 to 1 to each treatment. A higher SUCRA indicates a higher likelihood that a specific treatment is most suitable for the investigated outcome. Comparing the obtained SUCRA for different treatments permits the creation of a ranking among treatments. Where necessary, we converted the reported median and interquartile range to the estimated mean and standard deviation (SD) using the Hozo method [17].

When multiple local anesthetic dosing regimens were employed in the same study for the same block, the means and standard deviations were combined.

### Inconsistency, heterogeneity, and Publication bias analysis

For assessment of study heterogeneity, the Chi-squared test and I^2^-statistic were used (considering I^2^ values as follows: low: < 25%, moderate:25% to 50%, or high: > 50%) [18].Within design heterogeneity and between design inconsistency were evaluated using Cochrane’s Q. A random-effects model was preferred, regardless of both inconsistency and heterogeneity. Publication bias was evaluated by visual inspection of funnel plots and Egger’s test (p-value < 0.05, indicating a possible publication bias) (Supplementary Material 2).

## Results

### Study selection and data retrieval

**Fig. 1:**
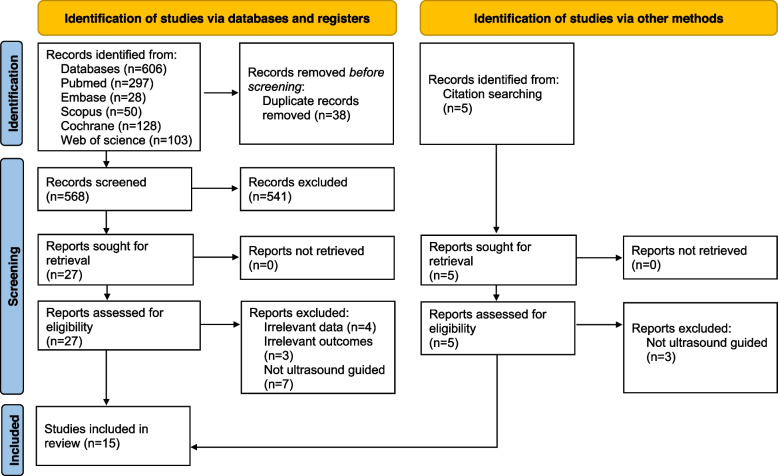
PRISMA flow diagram. The diagram shows the study selection process and provides reasons for exclusion for the records screened. PRISMA, Preferred Reporting Items for Systematic Reviews and Meta-Analyses

The search results are shown in the PRISMA diagram (Fig. 1). The initial screening identified 611 studies. Of these, 584 search results were excluded during the preliminary screening because they were unrelated or duplicated. The remaining 27 full-text manuscripts were retrieved, and a further 12 studies were excluded based on our inclusion and exclusion criteria. Fifteen studies evaluating four different regional anesthesia techniques were finally included for quantitative and qualitative analysis [19–33].

**Fig. 2:**
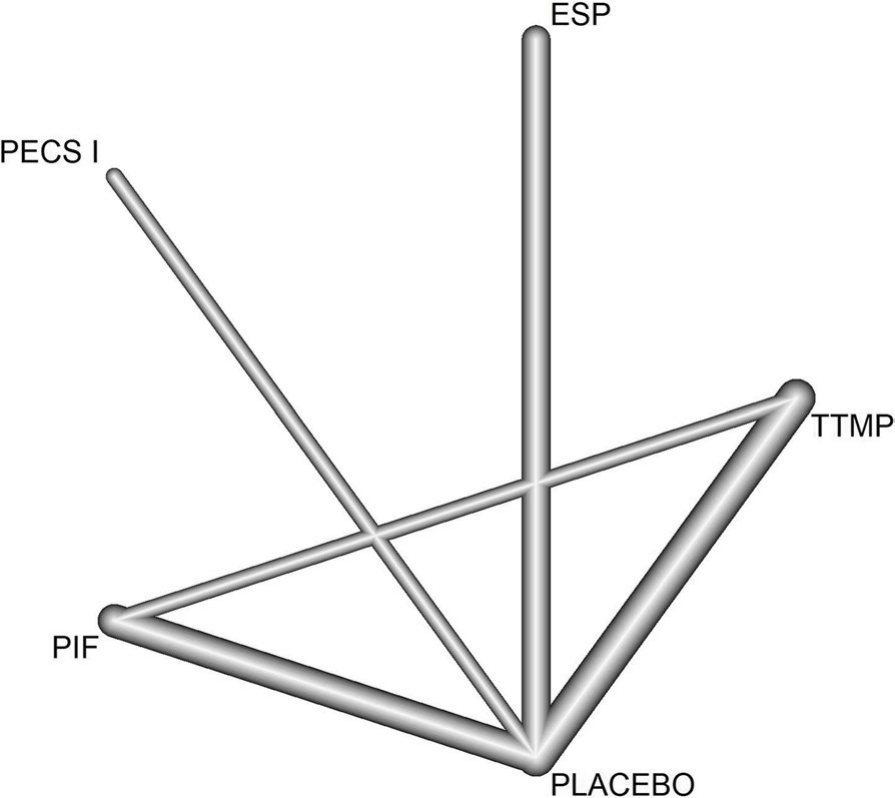
Network diagram comparing different regional anesthesia techniques in terms of intravenous morphine equivalents (mg) in the first 24 h

The regional and local anesthesia techniques included the pecto-intercostal fascial (PIF), transversus thoracis muscle plane (TTMP), erector spinae plane (ESP), and pectoralis nerve (PECS) I blocks ( Fig. 2).

The 15 included studies were randomized to 849 patients. Of these, 405 patients were allocated to placebo or no intervention, 183 to the PIF block, 148 to the TTMP block, 93 to the ESP block, and 20 to the PECS I block.

According to the risk of bias evaluation, four studies had a low risk of bias, two had a high risk of bias, and some concerns arose with the remaining studies (Supplementary material 3). The criteria that guided us in assigning the risk of bias judgments are available as supplementary material (Supplementary material 3).

## Outcomes

**Fig. 3:**
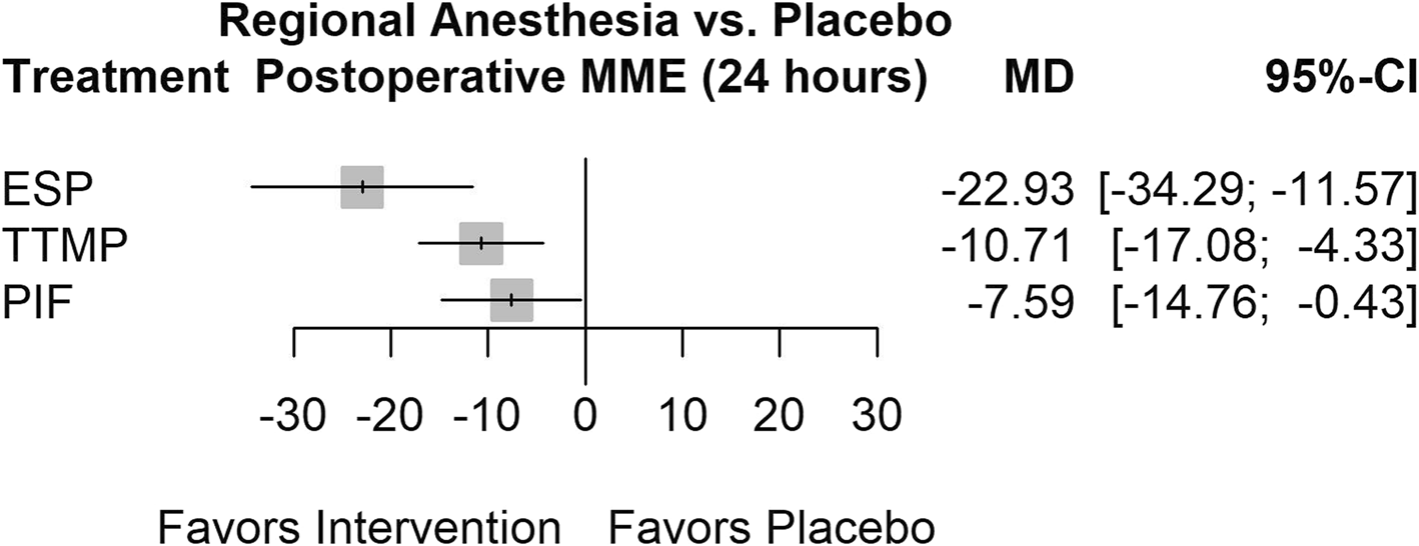
Forest plot for the comparison of intravenous morphine equivalents (mg) in the first 24 h. Comparison of results expressed in intravenous morphine equivalents (mg) through meand difference (MD) between subgroups

The results are graphically depicted in Figs. 2 and 3 for the main outcome and summarized in Table 1 for all outcomes; these results are based on the combination of direct and indirect evidence. Direct evidence is obtained from direct comparisons (e.g., A vs. B), while indirect evidence arises from indirect comparisons (e.g., A vs. B indirect evidence is provided by direct comparison of A vs. C and B vs. C).

**Table 1:** Summary of findings table

Author (Year)	Group 1	Group 2	Country	Age	ASA	LA1	LA2	GA protocol		PO analgesia protocol	Main Outcome
**Zhang Y (2021)** [19]	PIFB	PLACEBO	China	20–70	3–4	0.4% ropivacaine 20 ml	Saline 20 mL	INDUCTION: MDZ 0.05—0.1 mg/kg. SUF 0.8—1 μg/kg.; ETM 0.3 mg/kg and ROC 0.6 mg/kg MAINTENANCE: SUF. PRP and ROC		PCA SUF and 20 mg parecoxib iv at 6 h intervals	Postoperative pain
**Athar M (2021)** [20]	ESPB	PLACEBO	India	18–60		0.25% levobupivacaine 20 ml	Saline 20 mL	PREMEDICATION: diazepam 0.2 mg/kg INDUCTION: FNT 5 mcg/kg. ETM 0.3–0.6 mg/kg, ROC 0.6–1 mg/kg MAINTENANCE: SEVO 1–2%. FNT 5–10 mcg/kg/h			Fentanyl comsumption
**Kumar AK (2021)** [21]	PIFB	IV ANALGESIC	India	18–80		Ropivacaine 0.25% 10 ml	Saline 10 mL	INDUCTION: FNT. 2 mg/kg. THP 2–3 mg/kg. ROC 1 mg/kg. MAINTENANCE: isoflurane, FNT and ROC		Acetaminophen iv 1 g. and tramadol 50 mg every 6 h	Postoperative pain
**Khera T (2021)** [22]	PIFB	PLACEBO	USA	> 18		0.25% bupivacaine 20 ml	Saline 20 mL				Morphine comsumption
**Aydin ME (2020)** [23]	TTMPB	PLACEBO	Turkey		2–3	20 ml of 0.25% bupivacaine	Saline 20 mL	INDUCTION: MDZ 0.5 mg/kg; THP 5 mg/kg, ROC 0.6 mg/kg, REMI 0.5 mg/kg bolus MAINTENANCE: REMI 0.15—0.35 mg/kg/min		Tramadol 100 mg if VAS ≥ 4	Fentanyl comsumption
**Krishna SN (2019)** [24]	ESPB	IV ANALGESIC	India	20–70		3 mg/kg of 0.375% ropivacaine 20 to 25 ml	Not given	INDUCTION: FNT 1 mg/kg, MDZ 0.05 mg/kg, HP 3 -5 mg/kg, ROC 0.6 mg/kg MAINTENANCE: isoflurane and boluses of atracurium and FNT		Tramadol or FNT iv	Postoperative pain
**Chen H (2021)** [25]	PIFB	PLACEBO	China	18–65	1–2	Ropivacaine 0.5% 10 mL	Saline 10 mL	MAINTENANCE: SEVO and REMI. Cisatracurium		PCA-SUF 1.5—2.5 mcg/h and boluses of 1 mcg 10 min lockout interval (Max dose: 8 mcg/h)	Cumulative sufentanil consumption
**Hamed. M. A. (2022)** [26]	PIFB	PLACEBO	Egypt	> 18		Bupivacaine 0.25% 20 mL plus 5 mcg/mL epinephrine	Dry needle	INDUCTION: MDZ 2 mg, FNT 10 mcg/kg, PRP 2 mg/kg. atracurium 0.5 mg/kg MAINTENANCE: isoflurane 1%. atracurium 0.5 mg/kg		IV morphine 5–10 mg boluses (required)	Cumulative morphine consumption
**Kaya C (2021)** [27]	PIFB	TTMPB	Turkey	18–80	2–3	Bupivacaine 0.25% 20 mL plus 1:400,000 epinephrine	Bupivacaine 0.25% 20 mL plus 1:400,000 epinephrine	INDUCTION: MDZ 0.05–0.1 mg/kg, FNT 2–5 mcg/kg, pentothal 4–5 mg/kg. ROC 1 mg/kg MAINTENANCE: SEVO 1–2%. FNT 2–5 mcg/kg/h		Acetaminophen 1 g × 4. PCA: 20 mcg/kg morphine lockout time: 6–10 min, tramadol 100 mg (NRS > 3)	Morphine comsumption
**Shokri H (2021)** [28]	TTMPB	PLACEBO	Egypt	55–74	2–3	Bupivacaine 0.25% 15 mL	Saline 15 mL	PREMEDICATION: MDZ 1–3 mg INDUCTION: FNT 5–6 mcg/kg, PRP 2 mg/kg. ROC 0.6 mg/kg MAINTENANCE: isoflurane 1–2%		4 × 1gr paracetamol. morphine 0.05 mg/kg (VAS > 4)	Morphine comsumption
**Fujii S (2019)** [29]	TTMPB	IV ANALGESIC	Canada	18–90		Ropivacaine 0.3(weighing < 75 kg) -0.5 (> 75 kg) % 20 mL	No block	INDUCTION: PRP 0.5–1 mg/kg, MDZ 0.05–1 mg/kg, FNT 2–5 mcg/kg, ROC 0.6–1 mg/kg MAINTENANCE: isoflurane 0.5–1%		Hidmorphone, Acetaminophen, NSAID	Recruitment rate. Adherence rate. Adverse events rate
**Zhang (2021)** [30]	TTMPB	IV ANALGESIC	China	18–70	2–3	Ropivacaine 0.4% 20 mL	Saline 20 mL	INDUCTION: MDZ 0.1 mg/kg, ETM 0.3 mg/kg, cisatracurium 0.15 mg/kg, SUF 0.6–1 mcg/kg MAINTENANCE: PRP and cisatracurium		Sufentanyl iv additional pain: 4 × 50 mg flurbiprofen	Perioperative sufentanil consumption
**Kumar K (2018)** [31]	IV ANALGESİC	PECS I	India	25–65		No block	Bupivacaine 0.25% + dexmetedomidine 25 mcg 30 mL and bupivacaine 0.25% 5 mL			Paracetamol 1 gr + tramadol 50 mg iv + fentanyl 1 mcg/kg (VAS > 4 rescue analgesia) and diclofenac 75 mg (VAS > 4 30 min after first rescue analgesia)	Postoperative pain
**Hamed (2022)** [32]	TTMPB	PLACEBO	Egypt	> 18		20 mL of 0.25% bupivacaine	20 mL of 0.9% saline	PREMEDICATION: 10 mg morphine im INDUCTION: MDZ 2 mg, FNT (10 μg.kg − 1), PRP (3 mg.Kg − 1), and atracurium (0.5 mg.kg − 1) MAINTENANCE: Isoflurane 0.4%—1%, atracurium 0.5 mg/kg/h, PRP 50–100 μ/kg/min		IV FNT PCA with (10 μg/mL, bolus of 15 μg, lockout 10 min—MAX 90μ/hr). Acetaminophene 1 g every 8 h	Total fentanyl consumption
**Guven (2022)** [33]	ESPB	IV ANALGESIC	Turkey	18–65	1–2	20 ml of 0.25% bupivacaine	No block	PREMEDICATION: MDZ 0.03 mg/kg INDUCTION: FNT 1 µg/kg, MDZ 0.15 mg/kg, PRP 1–2 mg/kg, ROC 1 mg/kg. MAINTENANCE: SEVO, ROC infusion (0.01 mg/kg/min) and FNT (2–3 µg/kg/h)		Acetaminophen 1 g, 100 mg IV tramadol, IV PCA with morphine (0.5 mg/ml concentration, 1 mg bolus dose, lockout 10 min)	Total morphine consumption

*Abbreviations: PIFB* Pecto-intercostal fascial block, *MDZ* Midazolam, *SUF* Sufentanil, *ETM* Etomidate, *ROC* Rocuronium, *PRP* Propofol, *PCA* Patient-controlled analgesia, *ESPB* Erector spinae plane block, *FNT* Fentanil, *SEVO* Sevoflurane, *THP* Thiopenthal, *TTMPB* Transversus thoracis muscle plane block, *VAS* Visual analog scale, *NRS* Numerical rating scale, *NSAID* Non-steroidal anti-inflammatory drugs

### Primary outcome

#### Postoperative opioid consumption at 24 h

The postoperative opioid consumption was evaluated in 13 studies. There was an ESP block group in two studies, a PIF block group in six studies, and a TTMP block in five studies. No data were available on the PECS I block for this outcome.

All the RA techniques included were statistically significant in reducing postoperative opioid consumption at 24 h, expressed as morphine milligram equivalents (MME). The ESP block was the most effective treatment (-22.93 MME [-34.29;-11.56]). A forest plot of this outcome is shown in Fig. 3.

Using the GRADE assessment, the quality of evidence was rated low or very low (Table 2).

**Table 2:** GRADE assessment, the quality of evidence evaluated considering direct evidence, indirect evidence, and network meta-analysis evidence. Network meta-analysis evidence are calculated using the Bayesian method

Comparison	Direct evidence		Indirect evidence		Network meta-anaalysis	
	**SMD (95% CI)**	**Quality of evidence**	**SMD (95% CI)**	**Quality of evidence**	**SMD (95% CI)**	**Quality of evidence**
**ESPB versus placebo**	-	-	-22.93(-34.29;-11.57)	⊕ ⊕ ⊝ ⊝ **Low**	-22.93(-34.29;-11.57)	⊕ ⊕ ⊝ ⊝ **Low**
**PIFB versus placebo**	-6.57(-14.52;1.39)	⊕ ⊕ ⊝ ⊝ **Low**	-11.97(-28.41;4.46)	⊕ ⊝ ⊝ ⊝ **Very Low**	-7.59(-14.75;-0.44)	⊕ ⊕ ⊝ ⊝ **Low**
**TTMPB versus placebo**	-11.47 (-18.36;-4.59)	⊕ ⊕ ⊝ ⊝ **Low**	-6.07(-22.98;10.84)	⊕ ⊝ ⊝ ⊝ **Very Low**	-10.70(-17.08; -4.32)	⊕ ⊕ ⊝ ⊝ **Low**
**ESPB versus PIFB**	-	-	-15.34(-28.77;-1.91)	⊕ ⊝ ⊝ ⊝ **Very Low**	-15.34(-28.77;-1.91)	⊕ ⊝ ⊝ ⊝ **Very Low**
**ESPB versus TTMPB**	-	-	-12.23(-25.25;0.80)	⊕ ⊝ ⊝ ⊝ **Very Low**	-12.23(-25.25;0.80)	⊕ ⊝ ⊝ ⊝ **Very Low**
**PIFB versus TTMPB**	-0.50 (-15.42;14.42)	⊕ ⊕ ⊝ ⊝ **Low**	4.90(-5.61;15.42)	⊕ ⊝ ⊝ ⊝ **Very Low**	3.11(-5.48; 11.70)	⊕ ⊕ ⊝ ⊝ **Low**

*Abbreviations: GRADE* Grading of recommendations assessment, development, and evaluation, *SMD* Standardized mean difference, *ESPB* Erector spinae plane block, *PIFB* Pecto-intercostal fascial block, *TTMP* Transversus thoracis muscle plane block

### Secondary outcomes

#### Pain at 12 and 24 h

All the identified RA techniques were evaluated for this outcome.

Pain at 12 postoperative hours was evaluated for the ESP block in three studies, PIF block in four studies, TTMP block in five studies, and PECS I block in one study. Pain at 24 postoperative hours was evaluated in two studies with an ESP block group, in four studies with a PIF block group, in six studies with a TTMP block group, and one study with a PECS I block group. However, there were no statistically significant differences in any of these comparisons.

#### Time to extubation

This outcome was evaluated in nine studies. There was an ESP block group in two studies, a PIF block group in three studies, and a TTMP block in five studies. No data were available on the PECS I block for this outcome.

The PIF block was the only RA technique with statistically significant results; however, surprisingly, the required time to extubate the patient was longer for patients receiving this block 2.14 h (0.22 h to 4.06 h) when compared to placebo or no intervention.

#### Intensive care unit length of stay

Nine studies reported results for this outcome; two studies reported data for the ESP block, four studies for the PIF block, five studies for the TTMP block, and no data for the PECS I block.

Regarding intensive care unit (ICU) LOS, only the ESP block was significantly associated with a reduction in the LOS of -1.10 days (-2.01 -0.18).

#### Hospital length of stay

We only retrieved data for the PIF and TTMP blocks for hospital length of stay in comparison with placebo/no intervention. However, none of the RA techniques showed statistically significant differences.

#### Postoperative nausea and vomiting

Postoperative nausea and vomiting were evaluated for only six studies, and two studies reported this outcome for each block (ESP, PIF, and TTMP) but not the PECS I block. However, none of these RA techniques had a statistically significant result when compared with placebo.

## Dıscussıon

In this systematic review and NMA, we aimed to evaluate the effects of single-shot ultrasound-guided RA techniques on 24-h MME consumption in patients undergoing open cardiac surgery. Our NMA showed a statistically significant reduction in MMEs for the ESP, TTMP, and PIF blocks compared with placebo. No evidence was found for the PECS I block.

The efficacy of the ESP block in cardiac surgery has been extensively proven [24, 34]. There is evidence in the literature that this block has good analgesic effects in sternotomy cardiac surgery. In our NMA, we can infer that the ESP block has greater efficacy than placebo; compared with other blocks, it seems to be more effective than the PIF block. No differences were found between the ESP and TTMP blocks (Table 3). As in other fascial plane block studies, dermatomal analysis is not performed in studies evaluating the effectiveness of the ESP block, and the component of pain that is blocked has not been established. As mentioned before, pain in cardiac surgery may be caused by several factors, and the effectiveness of the ESP block may be due to blocking of the median sternotomy incision pain or acting in other ways. In future studies, questioning the exact location of pain and revealing the source by dermatome analysis will shed light on the blocks to be preferred and the combinations may provide postsurgical analgesia in cardiac sternotomy surgery.

**Table 3:**
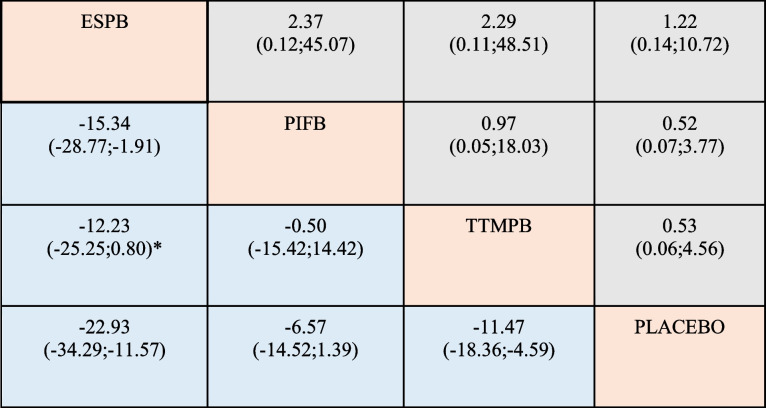
Direct and indirect effects of 24 h morphine consumption and postoperative nausea and vomiting and regional anesthesia compared with placebo

(*) Direct and indirect effects. In light blue; mean difference 24 h morphine milligram equivalents. In gray; odds ratio of postoperative nause and vomiting

*Abbreviations: ESPB* Erector spinae plane block, *PIFB* Pecto-intercostal fascial block, *TTMP* Transversus thoracis muscle plane block

The PIF and TTMP blocks are two new truncal fascial plane blocks that aim to anesthetize the anterior cutaneous branches of the thoracic intercostal nerves (Th2-6). Although these blocks have only recently been discovered, a 2021 ASRA-ESRA consensus renamed them superficial and deep parasternal intercostal plane blocks, respectively, to better define them anatomically [35]. It was shown to be effective in postoperative pain management in the context of cardiac sternotomy surgery [29]. The TTMP block in our NMA would seem to have a benefit in terms of 24-h MME consumption compared with placebo, not so evident is the benefit when compared directly to PIF block and indirectly to ESP block (Table 3). The PIF block is the first among these blocks to be described as effective in the literature regarding the use of sternotomy cardiac surgery [36]. In this NMA, its use seems to be the most questioned, as there does not appear to be a clear benefit in terms of reduction in MMEs when compared to placebo (Table 3).

When we evaluated the other outcomes, the PIF block seemed to be the only one to increase extubation time with a statistically significant result by increasing the time. This result is highly influenced by one study [19] that showed a high difference among the groups. In addition, a standard extubation protocol has not been used in the trials included in this NMA, and usually extubation protocols were determined by clinicians in ICU. This may affect the results. Also, the low sample size could have created a bias. Therefore, further evidence for this outcome is warranted.

The impact of the ESP and TTMP blocks on ICU LOS is significant and favorable. Another NMA that evaluates the effects of fascial blocks in cardiac surgery can be found in the literature, but this one does not discriminate between sternotomy and non-sternotomy procedures, and this is a major limitation, as these are quite different procedures in terms of postoperative pain compared to each other [37]. In-hospital LOS is assessed only for PIF and TTMP blocks and is not statistically significant.

While there are no clear data to define the minimal clinically important difference (MCID) for 24-h opioid consumption in the literature referring to sternotomy cardiac surgery procedures, it is difficult to determine the magnitude of the analgesic effect of fascial blocks in this population. Hussain et al. evaluated breast cancer operations and considered reductions equivalent to 10 mg i.v. morphine reduction to be clinically important [38]. Aware of the differences in the analgesic setting, considering the different structures involved in the surgical procedure, and comparing this to our results, we can assume that the ESP and TTMP blocks are effective.

In the present NMA, RA techniques were performed with amide type, long-acting local anesthetics (such as bupivacaine, levobupivacaine, and ropivacaine). Selecting a similar type of LAs may have resulted in comparable effects on the results. However, more randomized controlled trials on concentration, volume, and type of LA are necessary. In addition, only one study used dexmedetomidine as an adjuvant [31]. Although adjuvants are effective in peripheral nerve blocks, there is no clear data for improving the quality and prolonging the duration of analgesia in the fascial plane blocks. We attempted to relieve these effects by conducting subgroup analysis but failed because data were lacking.

This study has several limitations. First, the included studies are few, and most of them compared the blocks with placebo, making indirect comparison essential. In addition, publication bias assumed by Egger's test makes indirect comparison possible for 24 h MMEs, which is more difficult to estimate for other outcomes. Therefore, there is reduced consistency for untestable assumptions. Second, the heterogeneity in our analysis was very high. We attribute this to the fact that these blocks are relatively new, have been used in clinical settings in the last five years, and are being developed daily. Third, it should be specified that a placebo is often defined as an injection of saline instead of a local anesthetic, but sometimes studies represent placebo as no injection or medication. Fourth, the volume, type, and concentration of the local anesthetic administered varied. In some trials, adjuncts were added to the local anesthetic.

## Conclusions

Ultrasound-guided regional anesthesia is certainly a key analgesic technique in the context of cardiac surgery, as it spares opioids, reducing their neurological and hemodynamic impact but without interfering with the coagulative status of the patient, allowing its use in non-elective procedures. Although there are several fascial blocks, no single technique is better than the others. In this meta-analysis, we concluded that ESP, PIF, and TTMP blocks were better than placebo when evaluating 24-h MMEs. However, it is still challenging to determine which is better, given the lack of studies available in the literature. More high-quality RCTs are required to determine which regional anesthesia technique is better. An MCID should also be determined in cardiac surgery to quantify the effect of individual blocks compared with the standard of care.

## Supplementary Information

**Additional file 1: Supplementary material 1.** Search strategy.

**Additional file 2: Supplementary material 2.** Funnel plots for the postoperative opioid consumption at 24 hours.

**Additional file 3: Supplementary material 3.** Risk of bias assessment.

## Data Availability

The datasets used and/or analysed during the current study available from the corresponding author on reasonable request.

## Abbreviations

RA: Regional Anesthesia
NMA: Network Meta-Analysis
PONV: Postoperative Nausea And Vomiting
LOS: Length Of Stay
RCT: Randomized Controlled Trial
ROB: Risk Of Bias
MD: Mean Difference
CI: Confidence Interval
SUCRA: Surface Under The Cumulative Ranking Curve
PIF: Pecto-İntercostal Fascial
TTMP: Transversus Thoracis Muscle Plane
ESP: Erector Spinae Plane
PECS: Pectoralis Nerve
MME: Morphine milligram equivalents
ICU: Intensive care unit
