# Supplementary material for: Effects of ultrasound-guided regional anesthesia in cardiac surgery: a systematic review and network meta-analysis
Source: BMC Anesthesiol. 2022 Dec 29;22:409. doi: 10.1186/s12871-022-01952-7 (PMC9798577; doi:10.1186/s12871-022-01952-7)
Supplement: Supplementary file 1 — Additional file 1: Supplementary material 1. Search strategy. [file 12871_2022_1952_MOESM1_ESM.docx]

**Search Strategy**

| **#** | **Searches** | **Results** |
| --- | --- | --- |
| 1 | exp cardiac surgery | 553271 |
| 2 | exp (((((erector spinae plane block) OR (pectointercostal fascial plane block)) OR (transversus thoracic muscle plane block)) OR (parasternal intercostal nerve block)) OR (paravertebral block)) OR (local infiltration) | 45837 |
| 3 | 1 and 2 | 2051 |
| 4 | exp randomized controlled trial | 751193 |
| 5 | 3 AND 4 | 297 |

**Supplementary material 1.** Search strategy
