# Supplementary figures and images for: Effects of ultrasound-guided regional anesthesia in cardiac surgery: a systematic review and network meta-analysis
Source: BMC Anesthesiol. 2022 Dec 29;22:409. doi: 10.1186/s12871-022-01952-7 (PMC9798577; doi:10.1186/s12871-022-01952-7)

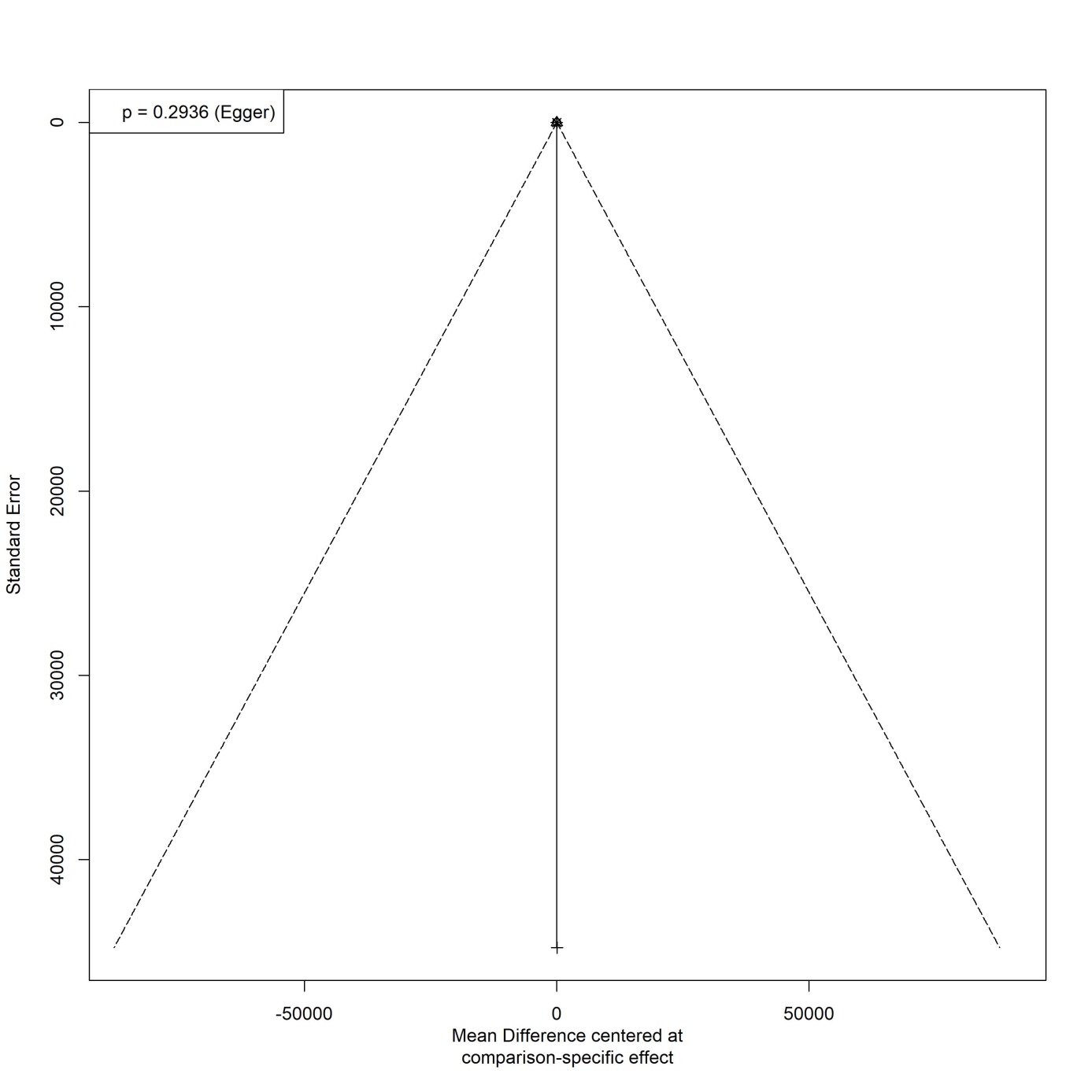


**Supplementary material 2.** Funnel plots for the postoperative opioid consumption at 24 hours.

Supplement: Supplementary file 2 — Additional file 2: Supplementary material 2. Funnel plots for the postoperative opioid consumption at 24 hours. [file 12871_2022_1952_MOESM2_ESM.docx]

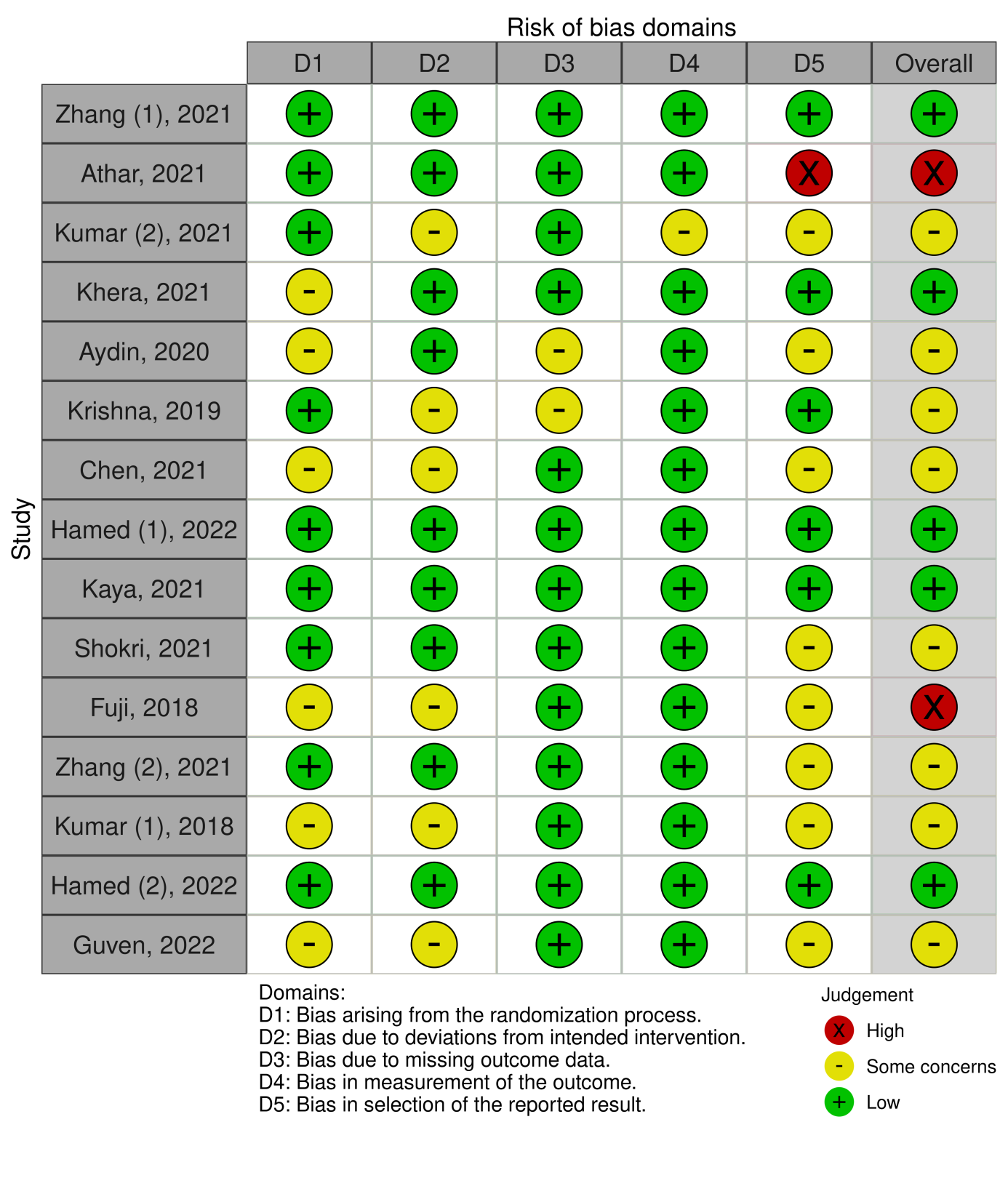


**Supplementary material 3.** Risk of bias assessment.

Supplement: Supplementary file 3 — Additional file 3: Supplementary material 3. Risk of bias assessment. [file 12871_2022_1952_MOESM3_ESM.docx]
